# Burden of Injuries Avertable By a Basic Surgical Package in Low- and Middle-Income Regions: A Systematic Analysis From the Global Burden of Disease 2010 Study

**DOI:** 10.1007/s00268-014-2685-x

**Published:** 2014-07-10

**Authors:** Hideki Higashi, Jan J. Barendregt, Nicholas J. Kassebaum, Thomas G. Weiser, Stephen W. Bickler, Theo Vos

**Affiliations:** 1 Institute for Health Metrics and Evaluation, University of Washington, 2301 Fifth Avenue, Suite 600, Seattle, Washington 98121 USA; 2 School of Population Health, University of Queensland, Brisbane, QLD Australia; 3 Division of Anesthesiology & Pain Medicine, Seattle Children’s Hospital, Seattle, Washington USA; 4 Department of Surgery, School of Medicine, Stanford University, Stanford, California USA; 5 Department of Surgery, School of Medicine, University of California, San Diego, California USA

## Abstract

**Background:**

Injuries accounted for 11 % of the global burden of disease in 2010. This study aimed to quantify the burden of injury in low- and middle-income countries (LMICs) that could be averted if basic surgical services were made available and accessible to the entire population.

**Methods:**

We examined all causes of injury from the Global Burden of Disease 2010 Study. We split the disability-adjusted life years (DALYs) for these conditions between surgically “avertable” and “nonavertable” burdens. For estimating the avertable fatal burden, we applied the lowest fatality rates among the 21 epidemiologic regions to each LMIC region, assuming that the differences in death rates between each region and the lowest rates reflect the gap in surgical care. We adjusted for fatal cases that occur prior to reaching hospitals as they are not surgically avertable. Similarly, we applied the lowest nonfatal burden per case to each LMIC region.

**Results:**

Overall, 21 % of the injury burden in LMICs was potentially avertable by basic surgical care (52.3 million DALYs). The avertable proportion was greater for deaths than for nonfatal burden (23 vs. 20 %), suggesting that surgical services for injuries more effectively save lives than ameliorate disability. Sub-Saharan Africa had the largest proportion of potentially avertable burden (25 %). South Asia had the highest total avertable DALYs (17.4 million). Road injury comprised the largest total avertable burden in LMICs (16.1 million DALYs).

**Conclusions:**

Basic surgical care has the potential to play a major role in reducing the injury-related burden in LMICs.

**Electronic supplementary material:**

The online version of this article (doi:10.1007/s00268-014-2685-x) contains supplementary material, which is available to authorized users.

## Introduction

The Global Burden of Diseases, Injuries, and Risk Factors (GBD) 2010 Study reported that 5 million deaths and 279 million disability-adjusted life years (DALYs) globally were due to injuries in 2010 [1]. Injuries accounted for 11 % of the total disease burden globally (2,490 million DALYs) in both high-income countries (HICs) and low- and middle-income countries (LMICs). Although the proportion of injury-related burden to the total burden was virtually identical between HICs and LMICs in 2010, there were some notable differences. First, there was a declining trend since 1990 in the proportion of burden due to injuries in HICs but an increasing trend in LMICs. Second, although road injury, falls, and self-harm were the top three causes of injury burden globally, their relative importance differed. These three causes contribute more or less equally to the injury burden in HICs (25, 26, and 21 %, respectively), whereas road injuries clearly dominate in LMICs (27, 11, and 12 %, respectively). Drowning, fire, heat, and hot substances account for 15 % of the injury burden in LMICs, whereas these factors are responsible for only 5 % of injuries in HICs.

Surgically avertable burden for all conditions (not just injury) was estimated for the first time as part of the Disease Control Priorities Project, Version 2 (DCP2) [2]. Opinions from 18 surgeons were elicited, and their responses estimated that 11 % of the global disease burden might be addressed by surgical care. Also, 38 % of that burden was deemed to be related to injuries [3]. Given the recent release of GBD 2010 Study estimates and enhanced methods for disease burden in general, we are now in the position to examine the surgically avertable burden by applying a more data-driven approach. In the current study, we focused on all injuries (the largest contributor to the global surgical burden) that are amenable to surgery in LMICs. Our aim was to quantify the burden of injury in LMICs that could be averted if a basic package of appropriate quality surgical services was universally available and accessible to the entire population. We incorporated data, methods, and resources of the GBD 2010 Study to arrive at the most comprehensive estimates of surgical burden of injuries to date.

## Methods

We examined all injury causes that were included in GBD 2010 and are listed in Table 1 [1]. Our analysis assumes a basic surgical package (also outlined in Table 1) that was identified based on priority recommendations from guidelines [4, 5]. These procedures can be provided at first-level hospitals and thus have the potential for scaling up even in rural areas. Conversely, we did not include advanced level surgical procedures (e.g., repair of major vascular injuries) that are more difficult to expand nationwide. The overall concept of the approach was to split the reported DALYs of injury in 2010 into surgically “avertable burden” and surgically “nonavertable burden” categories.

**Table 1:** Categories of injury and basic surgical services included in the estimation of surgically avertable burden in LMICs

Cause of injury	Surgical services
Road injury	Basic resuscitation
Other transport injury	Surgical airway
Falls	Peripheral venous access
Fire, heat, and hot substances	Suturing
Unintentional injury others^a^	Laceration and wound management
Interpersonal violence	Chest tube/needle decompression
Self-harm	Fracture reduction
Poisoning	Escharotomy
Drowning	Fasciotomy
Intentional injury others^b^	Skin grafting
	Trauma-related amputation
	Trauma-related laparotomy

*LMICs* low- and middle-income countries

^a^Includes exposure to mechanical forces, adverse effects of medical treatment, animal contact (venomous/nonvenomous), and unintentional injuries not classified elsewhere from the GBD 2010 Study

^b^Includes exposure to forces of nature, collective violence and legal intervention

We first estimated the DALYs that would remain if surgical coverage were scaled up to the “counterfactual” state of full access to quality surgical care such as what we expect in high-resource environments, thus leaving only the nonavertable burden. We then subtracted the nonavertable burden from the burden reported in the GBD 2010 to derive the avertable burden. Some of the external causes were assumed not amenable to surgery: drowning, poisoning, self-harm, venomous animal contact, intentional injury others. Key demographic and epidemiologic parameters for the analysis were obtained from GBD 2010, including population, standard life expectancy, cause-specific mortality, incidence, prevalence, and disability weight [6–8]. Most of the parameters were specific to age, sex, region, and year. The GBD 2010 grouped the countries into 21 epidemiologic regions and 7 super regions (6 of which are LMICs). Our analysis was conducted at the super-region level by aggregating regional-level parameters.

We estimated the fatal and nonfatal burdens separately: years of life lost (YLLs) and years lived with disability (YLDs). In GBD 2010, YLLs were estimated based on external causes of injury and YLDs based on the nature of the injury. The estimated YLDs based on nature of injury were then apportioned to each external cause based on cause-nature matrices to calculate the DALYs for each external cause. The details of this methodology have been previously described [7]. In this study, we estimated the avertable YLLs and YLDs based on external causes and nature of injury, respectively, and proportionally allocated the latter back to external causes. The proportional allocation of YLDs, instead of using a conversion matrix, was because of the absence of such matrices for surgically avertable burden.

The base population for the analysis comprised all incident cases in 2010. We initially estimated the number of deaths for each cause in LMIC super-regions if surgical coverage had been provided at the counterfactual state. We assumed that the epidemiologic region with the lowest age- and sex-specific case fatality rates (CFRs) for each cause reflected the counterfactual situation (hence the difference in those fatality rates reflecting the gap in surgical care). However, there are injury cases that result in deaths before reaching a hospital. As these cases are not amenable to surgical care, the counterfactual CFRs had to be adjusted for differences in proportions reaching the hospital alive. We obtained therefore information by surveying the literature [9–38] and country-level hospital data (Brazil, United States, South Africa) from a total of 18 countries on the proportion of prehospitalization deaths. We pooled and extrapolated those data to regions where there was no information available by means of Bayesian meta-regression embedded in the DisMod-MR program (see Vos et al. [7] for details on DisMod-MR). With this proportion of fatal prehospitalization cases, we adjusted the CFRs for each super-region (see Online Appendix for details) and estimated the fatal burden for the counterfactual state as follows:

1 $${\text{DEATH}}_{i,j,k}^{'} = {\text{Incidence}}_{i,j,k} \times {\text{CFR}}_{i,j,k}^{'}$$

2 $${\text{YLL}}_{i,j,k}^{'} = {\text{DEATH}}_{i,j,k}^{'} \times {\text{StdLifeExpect}}_{i}$$

where ${\text{DEATH}}_{i,j,k}^{'}$ is the age (*i*)- and sex (*j*)-specific number of deaths for the counterfactual state in each super-region (*k*), ${\text{Incidence}}_{i,j,k}$ is the age- and sex-specific number of incident cases from GBD 2010 in each super-region, ${\text{CFR}}_{i,j,k}^{'}$ is the lowest age- and sex-specific CFRs from all regions adjusted for prehospitalization deaths, ${\text{YLL}}_{i,j,k}^{'}$ is the fatal burden in the counterfactual situation, and ${\text{StdLifeExpect}}_{i}$ is the age-specific standard life expectancy used in GBD 2010 [6, 39].

The next step was to estimate the nonfatal burden for the counterfactual state. In GBD 2010, the incidence was initially calculated by external causes of injury. However, the disability weights were specific to the nature of the injury. Hence, the incidence estimates based on external causes were mapped to 23 natures of injury using cause–nature matrices that were age/sex-specific and distinguished between HICs and LMICs. (The matrices were constructed using hospital discharge data from more than 30 countries and emergency room discharge data from the United States, providing data both as external cause and nature for the same case.) The incidence by nature was the basis for calculating the short-term YLDs for each condition. For estimating the long-term YLDs, the prevalence of each condition was estimated by DisMod-MR using the estimated proportions of long-term sequeleae and incidence data based on the nature of injury. When estimating the total YLDs (i.e., short-term and long-term) for the counterfactual state, information of proportion resulting in long-term sequelae with optimal care was needed. As such information was not available, we assumed that the lowest total YLDs per case warranting access to care from all regions in GBD 2010 reflected the counterfactual situation. Based on these assumptions, the total YLDs based on natures of injury for the counterfactual state were calculated as:

3 $${\text{YLD}}_{i,j,k}^{'} = {\text{YLD}}_{i,j,k} \times {\text{YLDperCase}}_{i,j,k}^{\text{lowest}}$$

where ${\text{YLD}}_{i,j,k}^{'}$ is the nonfatal burden in case of the counterfactual state, and ${\text{YLDperCase}}_{i,j,k}^{\text{lowest}}$ is the lowest YLDs per case based on nature of injury warranting access to care from all regions. We then assigned back the avertable YLDs to each external cause by calculating the proportion of total YLDs that were avertable in each super-region and applied that proportion to GBD 2010 results that were provided as external causes. To account for external causes that were assumed to be not amenable to surgical care, we redistributed those YLDs to surgically amenable external causes proportionally and derived the final YLDs for the counterfactual state for each cause that was amenable to surgical care (see Online Appendix for details).

## Results

**Fig. 1:**
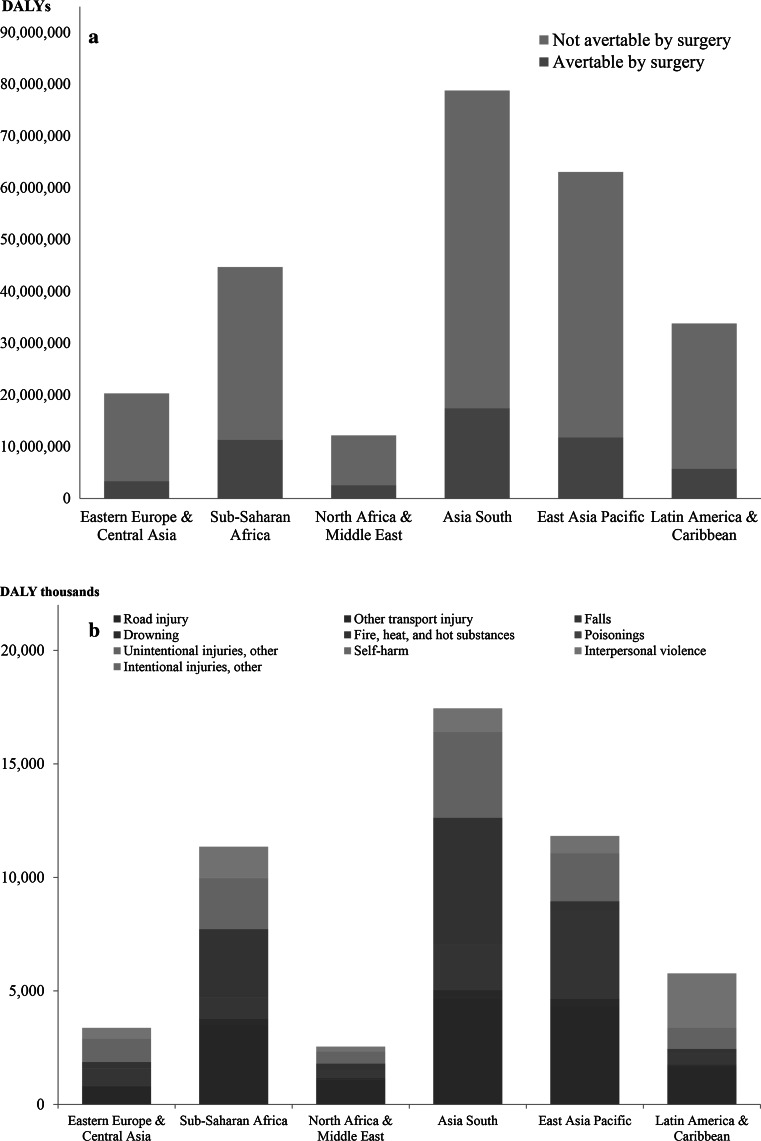
**a** Disability-adjusted life years (DALYs) by low- and middle-income (LMIC) super-region for all injuries combined. **b** Breakdown of surgically avertable burden in each LMIC super-region by cause of injury

A total of 52.3 million injury DALYs in 2010 could have been prevented if a basic package of quality surgical services in LMICs were fully available. This represented 21 % of the total injury burden in these countries. Figure 1a shows the surgically avertable and nonavertable burdens broken down by LMIC region. Sub-Saharan Africa had the largest proportion of potentially avertable burden (25 %), and South Asia had the highest total number of avertable DALYs (17.4 million) among the LMIC super-regions. Eastern Europe/Central Asia and Latin America/Caribbean had the lowest proportions of avertable burden (17 % each), and North Africa/Middle East the lowest total surgically avertable DALYs (2.5 million).

Figure 1b further quantifies the surgically avertable burden in each super-region by external causes of injury. Road injuries were the top cause of avertable burden in three super-regions (sub-Saharan Africa, North Africa/Middle East, East Asia Pacific). South Asia had more surgically avertable DALYs due to road injury than any other region (26 % surgically avertable, 4.7 million DALYs), but it ranked second in the region following injuries due to fire, heat, and hot substances (59 % surgically avertable, 5.6 million DALYs). In Latin America/Caribbean, interpersonal violence was the top cause of injury-related burden (30 % surgically avertable, 2.4 million DALYs), which was 40.8 % higher than the burden due to road injuries (29 % avertable, 1.7 million DALYs). Other unintentional injuries—a category that includes exposure to mechanical forces, adverse effects of medical treatment, animal contact, and unintentional injuries not classified elsewhere—had the highest proportion of surgically avertable burden in Eastern Europe/Central Asia (28 %, 1.0 million DALYs).

**Fig. 2:**
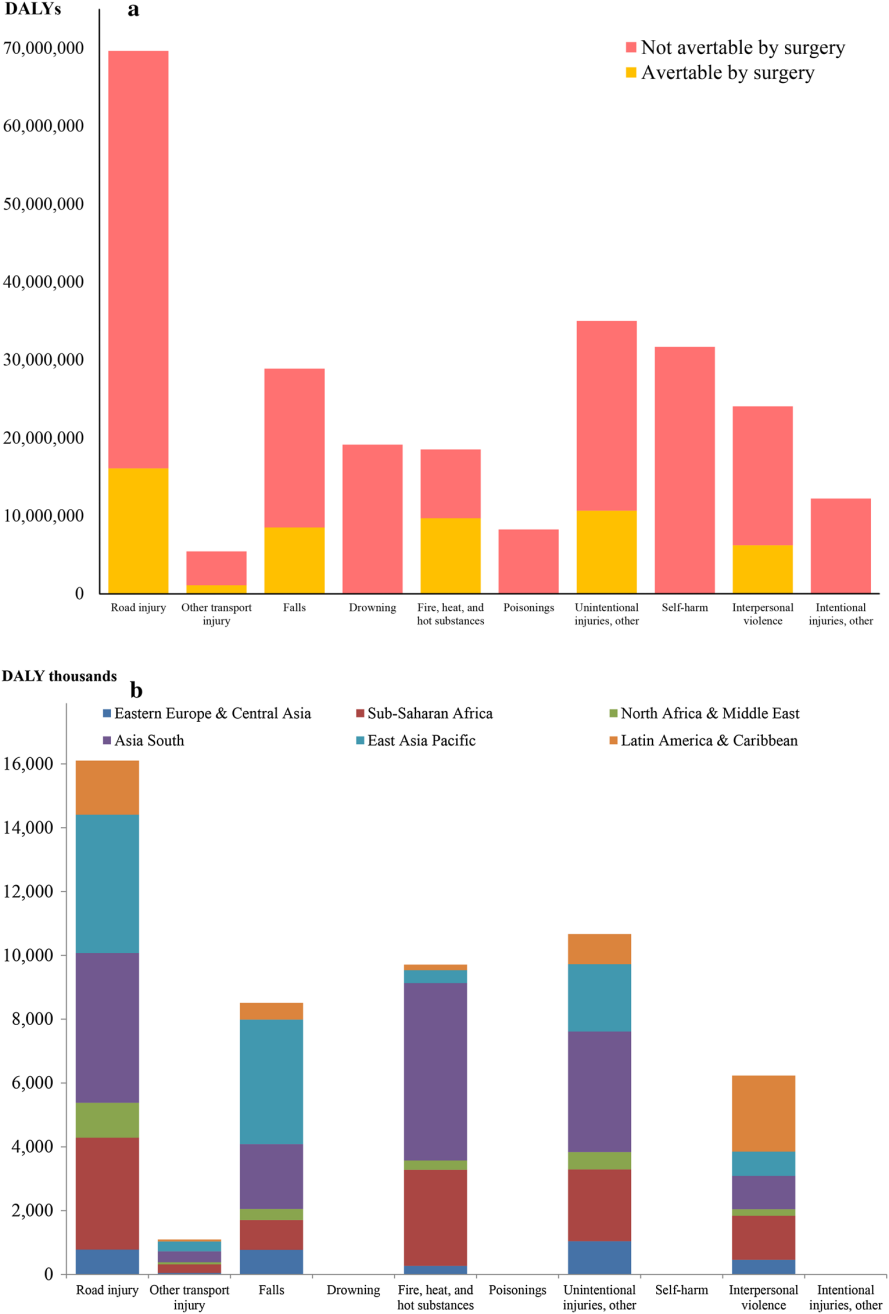
**a** DALYs by cause of injury for all LMIC super regions combined. **b** Breakdown of surgically avertable burden for each cause of injury by LMIC super-region

Figure 2a separates the global injury burden by external causes. Road injury comprised the largest avertable burden in LMICs (16.1 million DALYs). This avertable proportion was similar to the global total for all injuries (23 %). Injuries due to fire, heat, and hot substances comprised the largest proportion of potentially avertable burden (52 %, 9.7 million DALYs), followed by “other” unintentional injuries (30 %, 10.7 million DALYs) and falls (29 %, 8.5 million DALYs). Figure 2b further breaks down the avertable injury burden for each injury cause by region.

South Asia stands out in four categories of injury in terms of quantity of surgically avertable burden: injuries due to fire, heat, and hot substances; road injury; other transport injury; unintentional others. East Asia Pacific had the highest avertable DALYs caused by falls and Latin America/Caribbean had the highest caused by interpersonal violence. Table 2 provides full details of our estimates for surgically avertable deaths, YLLs, YLDs, and DALYs by super-regions and causes of injury. The avertable proportion is greater for deaths than for YLDs (23 vs. 20 %), suggesting that surgical services for injuries more effectively save lives than ameliorate disability.

**Table 2:** Injury-induced burden of deaths, YLLs, YLDs, and DALYs avertable by a basic package of quality surgical services in LMIC super-regions, by cause of injury

Outcomes^*a*^	East Europe/Central Asia	Sub-Saharan Africa	North Africa/Middle East	South Asia	East Asia Pacific	Latin America/Caribbean	Total
Road injury							
Death	14,342	59,218	19,832	86,610	76,976	34,894	291,872
	(19 %)	(26 %)	(25 %)	(28 %)	(18 %)	(32 %)	(24 %)
YLL	655,588	3,329,049	943,499	3,836,856	3,338,636	1,578,955	13,682,584
	(20 %)	(26 %)	(25 %)	(26 %)	(18 %)	(31 %)	(24 %)
YLD	123,720	178,589	149,622	856,142	998,241	112,358	2,418,673
	(11 %)	(16 %)	(15 %)	(26 %)	(24 %)	(13 %)	(21 %)
DALY	779,308	3,507,638	1,093,122	4,692,999	4,336,877	1,691,314	16,101,257
	(18 %)	(25 %)	(23 %)	(26 %)	(19 %)	(29 %)	(23 %)
Other transport injury							
Death	405	3,618	633	3,325	2,220	715	10,916
	(6 %)	(24 %)	(14 %)	(25 %)	(14 %)	(16 %)	(18 %)
YLL	20,922	232,883	33,867	165,347	104,157	34,114	591,289
	(7 %)	(26 %)	(14 %)	(25 %)	(15 %)	(17 %)	(20 %)
YLD	26,199	37,803	32,842	175,413	205,772	25,011	503,039
	(11 %)	(16 %)	(15 %)	(26 %)	(24 %)	(13 %)	(21 %)
DALY	47,120	270,686	66,709	340,760	309,928	59,125	1,094,328
	(9 %)	(24 %)	(15 %)	(26 %)	(20 %)	(15 %)	(20 %)
Falls							
Death	18,731	15,823	3,224	35,239	95,405	15,148	183,570
	(54 %)	(27 %)	(31 %)	(25 %)	(62 %)	(55 %)	(43 %)
YLL	568,149	758,9 73	179,756	1,109,891	2,586,471	366,664	5,569,904
	(60 %)	(26 %)	(39 %)	(21 %)	(61 %)	(56 %)	(38 %)
YLD	204,660	175,235	167,069	921,802	1,315,749	157,374	2,941,888
	(11 %)	(16 %)	(15 %)	(26 %)	(24 %)	(13 %)	(20 %)
DALY	772,809	934,208	346,824	2,031,692	3,902,220	524,038	8,511,792
	(27 %)	(23 %)	(22 %)	(23 %)	(40 %)	(28 %)	(29 %)
Fire, heat, and hot substance injuries							
Death	6,772	44,754	5,016	104,373	7,583	3,922	172,421
	(37 %)	(53 %)	(47 %)	(60 %)	(30 %)	(42 %)	(53 %)
YLL	257,650	2,983,578	275,890	5,458,924	316,613	159,126	9,451,781
	(40 %)	(53 %)	(51 %)	(60 %)	(33 %)	(44 %)	(55 %)
YLD	12,818	27,082	15,740	100,145	86,562	14,558	256,905
	(11 %)	(16 %)	(15 %)	(26 %)	(24 %)	(13 %)	(21 %)
DALY	270,469	3,010,660	291,629	5,559,069	403,174	173,684	9,708,685
	(35 %)	(52 %)	(45 %)	(59 %)	(31 %)	(36 %)	(52 %)
Unintentional injury, others^*b*^							
Death	40,740	48,668	15,256	86,689	56,684	26,692	274,728
	(50 %)	(42 %)	(50 %)	(41 %)	(39 %)	(57 %)	(44 %)
YLL	990,797	2,194,026	494,652	3,533,668	1,789,171	875,082	9,877,398
	(30 %)	(31 %)	(31 %)	(34 %)	(27 %)	(43 %)	(32 %)
YLD	51,199	57,143	47,487	244,439	322,145	68,433	790,846
	(10 %)	(15 %)	(14 %)	(25 %)	(23 %)	(12 %)	(19 %)
DALY	1,041,996	2,251,169	542,139	3,778,108	2,111,317	943,515	10,668,244
	(28 %)	(31 %)	(28 %)	(33 %)	(27 %)	(36 %)	(30 %)
Interpersonal violence							
Death	9,905	21,997	3,261	16,723	12,557	44,342	108,784
	(23 %)	(25 %)	(23 %)	(25 %)	(19 %)	(29 %)	(25 %)
YLL	448,054	1,356,085	187,016	938,030	642,464	2,367,070	5,938,719
	(23 %)	(26 %)	(25 %)	(26 %)	(20 %)	(30 %)	(26 %)
YLD	13,560	22,617	19,008	105,330	119,448	13,957	293,920
	(11 %)	(16 %)	(15 %)	(26 %)	(24 %)	(13 %)	(21 %)
DALY	461,613	1,378,702	206,024	1,043,360	761,912	2,381,028	6,232,639
	(23 %)	(26 %)	(23 %)	(26 %)	(20 %)	(30 %)	(26 %)
Injury totals							
Death	90,894	194,078	47,222	332,960	251,425	125,714	1,042,292
	(23 %)	(27 %)	(26 %)	(23 %)	(21 %)	(21 %)	(23 %)
YLL	2,941,160	10,854,594	2,114,680	15,042,717	8,777,512	5,381,012	45,111,674
	(18 %)	(26 %)	(23 %)	(22 %)	(18 %)	(18 %)	(21 %)
YLD	432,156	498,470	431,767	2,403,271	3,047,916	391,692	7,205,271
	(10 %)	(15 %)	(14 %)	(25 %)	(23 %)	(12 %)	(20 %)
DALY	3,373,316	11,353,064	2,546,447	17,445,988	11,825,428	5,772,703	52,316,945
	(17 %)	(25 %)	(21 %)	(22 %)	(19 %)	(17 %)	(21 %)

Percentages in parentheses reflect the avertable proportions

*YLL* years of life lost, *YLD* years lived with disability, *DALYs* disability-adjusted life years

^a^Note that poisoning, drowning, self-harm, and intentional injury, others (includes exposure to forces of nature, collective violence, legal intervention) were assumed not avertable and so are not included in the table

^b^Includes exposure to mechanical forces, adverse effects of medical treatment, animal contact (venomous/nonvenomous), and unintentional injuries not classified elsewhere in the GBD 2010 Study

## Discussion

Our analysis revealed that 1.0 million deaths and 52.3 million DALYs could have been averted if coverage for a basic surgical package were to be scaled up, with high-quality care reaching the entire population of LMICs in 2010. These cases account for 2.3 % of total burden of disease and injury in LMICs. The largest opportunity for survival was identified for those injured by fire, heat, and hot substances (53 % of deaths, 52 % of DALYs), which may be explained, to some extent, by the relatively small proportion of prehospitalization fatal cases in this category (male 43 %, female 37 %). Although falls have similarly low proportions of prehospitalization death (male 38 %, female 36 %) and a relatively high chance of survival after surgical care (43 %), the avertable burden is limited (29 %). This is largely due to the nature of falls, which affect primarily the older population for whom the avertable YLLs from prevented deaths are essentially lower compared to the younger population.

A comparison of our findings with other, similar studies would be worth noting. Mock et al. estimated the avertable deaths due to injury in LMICs, suggesting 1.73 million deaths to be avertable in 2008 [40], which is higher than our estimation (1.0 million deaths). They made use of CFRs from injuries obtained from three economic strata in their analysis: 35 % from Seattle, USA (high income); 55 % from Monterrey, Mexico (middle income); 63 % from Kumasi, Ghana (low income). These three CFRs were uniformly applied to all 2008 trauma mortality data in LMICs. Although the study is not immediately comparable because of differences in study years, data sources, and assumptions employed, our analysis was based on the most comprehensive and internally consistent sets of epidemiologic parameters to date from the GBD 2010 Study. They were specific to sex, age, region, and cause of injuries and hence ought to be more accurate.

When comparing our estimate of the proportion of surgically avertable injury burden with that reported in the Disease Control Priorities Project, Version 2 [3], our estimate was lower (35 vs. 21 %). In our analysis, we explicitly accounted for the proportion of deaths that occur on site or never reach a hospital (see Online Appendix), which might partly explain the difference. For instance, more than one in two deaths from road injuries and two in three deaths from other transport injuries or interpersonal violence took place before reaching the first referral point of care. The chance of survival for these cases depended largely on first aid and referral systems, which are much broader infrastructural and policy issues and hence cannot be addressed by surgical care alone. Nonetheless, the main driver of the difference comes from the approach that is considerably more systematic than the one employed in DCP2, which was based on expert opinion.

As is the case with any modeling study, our analysis is not without limitations. We assumed that the lowest fatality estimates from the 21 epidemiologic regions, adjusted for preadmission death, reflected the case of the counterfactual state of complete access to high-quality surgical care. Although most of the lowest age- and sex-specific fatality rates were from high-income regions, it is not evident if those figures reflect our counterfactual state in terms of what is practical in LMICs. Furthermore, even the lowest fatality rate may not truly reflect the case of full coverage with access to quality care. The variations in fatality rates among HICs may imply that none of the health systems reflect the true counterfactual state. Analysis of avertable YLDs was conducted at the level of the nature of injury categories that were pooled and reallocated proportionally to external causes. This condition was due to the absence of a conversion matrix between external causes and natures of injury. Although it does not affect the total YLDs of injury, there may be an over- or under-allocation of avertable nonfatal burden between external causes. Given the dominance of YLL over YLD in the total and avertable burden of injury, the impact of the proportional YLD allocation on results was minimal. Finally, it should be noted that the difficulties associated with scaling up surgical care can vary substantially between regions and procedures. Studies from South Africa, an upper-middle-income country, revealed major lack of trauma care system in the country where more than half the doctors at district hospitals do not have access to an operating room for emergency trauma cases, and 70 % are not able to perform laparotomy independently [41, 42].

## Conclusions

A substantial number of deaths can be averted—and disability addressed—with improvements in access to and delivery of basic surgical care in LMICs. There is considerable scope for surgical care to play in averting the burden of injury in LMICs. Despite the importance of surgical care, other approaches are needed to alleviate injury burden. Given the substantial proportion of fatal cases that occur prior to admission to hospitals, prevention and improvements in prehospitalization services are critical.

## Electronic supplementary material

Below is the link to the electronic supplementary material.

Supplementary material 1 (PDF 278 kb)
