# Supplementary material for: Burden of Injuries Avertable By a Basic Surgical Package in Low- and Middle-Income Regions: A Systematic Analysis From the Global Burden of Disease 2010 Study
Source: World J Surg. 2014 Jul 10;39(1):1–9. doi: 10.1007/s00268-014-2685-x (PMC4273085; doi:10.1007/s00268-014-2685-x)
Supplement: Supplementary file 1 — Supplementary material 1 (PDF 278 kb) [file 268_2014_2685_MOESM1_ESM.pdf]

Online Appendix

Burden of injuries avertable by a basic surgical package in low and middle-income regions: a systematic analysis from the Global Burden of Disease 2010 Study

Hideki Higashi, PhD[a,b,*], Jan J. Barendregt, PhD[b], Nicholas J. Kassebaum, MD[a,c], Thomas G. Weiser, MD[d], Stephen W. Bickler, MD[e], Theo Vos, PhD[a,b]

[a]Institute for Health Metrics and Evaluation, University of Washington, Seattle, WA, USA
[b]School of Population Health, University of Queensland, Brisbane, QLD, Australia
[c]Division of Anesthesiology & Pain Medicine, Seattle Children's Hospital, Seattle, WA, USA
[d]Department of Surgery, School of Medicine, Stanford University, Stanford, CA, USA
[e]Department of Surgery, School of Medicine, University of California, San Diego, CA, USA

*Correspondence: h.higashi@uqconnect.edu.au

1 Detailed methods

1.1 Adjustment of case fatality rates

In order to account for the fatal cases that occur before reaching a hospital, we adjusted the case-fatality rates (CFRs) for each super region by applying the proportions estimated in the eTable as follows:

$$CFR'_{i,j,k} = CFR_{i,j,k} \times NotReachProp_k + CFR^{lowest}_{i,j,k} \times (1 - NotReachProp_k)$$

where $CFR_{i,j,k}$ is the age (*i*) and sex (*j*) specific CFRs from each epidemiological region, $CFR'_{i,j,k}$ is the lowest age and sex-specific case-fatality rates (CFRs) from all regions adjusted for pre-hospital deaths, $NotReachProp_k$ is the proportion of fatal cases that take place before reaching hospitals in each region.

1.2 Assigning back avertable YLDs to external causes

In assigning back the avertable YLDs based on natures of injury to each external cause, we calculated the proportion of total YLDs that are avertable in each super region, and applied that proportion to GBD 2010 results that were provided as external causes:

$$YLDavert_{e,k} = YLD_{e,k} \times \frac{YLD_{E,k} - YLD'_N}{YLD_{E,k}}$$

where $YLDavert_{e,k}$ is the all age and sex YLDs for each external cause and super region that are potentially avertable, $YLD_{e,k}$ the all age and sex YLDs for each external cause in each super region from GBD 2010, $YLD_{E,k}$ the all age and sex YLDs summed over all external causes in each super region from GBD 2010, and $YLD_{N,k}$ the all age and sex YLD summed over all natures of injury in each super region in case of the counterfactual state

1.3 Redistribution of avertable YLDs to surgically amenable causes

In order to account for external causes that were assumed to be not amenable to surgical care, we redistributed those avertable YLDs to surgically amenable external causes proportionally and derived the final YLDs for the counterfactual state for each cause that is amenable to surgical care as follows:

$$YLD'_{e,k} = YLD_{e,k} - \left(YLDavert_{ea,k} + YLDavert_{En,k} \times \frac{YLDavert_{ea,k}}{YLDavert_{Ea,k}}\right)$$

where $YLDavert_{ea,k}$ is the avertable YLDs allocated to each external cause assumed to be surgically amenable in each region, $YLDavert_{En,k}$ the sum of avertable YLDs that were temporary allocated to external causes assumed to be surgically not amenable in each region, and $YLDavert_{Ea,k}$ the sum of avertable YLDs allocated to external causes assumed to be surgically amenable in each region.

eTable. Average cause-specific proportions of fatal cases prior to reaching hospitals

	East Europe & Central Asia	Sub-Saharan Africa	North Africa & Middle East	Asia South	East Asia Pacific	Latin America & Caribbean	**Total**
**Male**							
**Road injury**	54%	60%	49%	46%	55%	51%	**54%**
**Other transport injury**	75%	72%	71%	60%	72%	71%	**72%**
**Falls**	26%	55%	36%	65%	34%	30%	**38%**
**Fire, heat and hot substances**	47%	45%	33%	36%	43%	43%	**43%**
**Unintentional injury others**							
Exposure to mechanical forces	63%	72%	59%	59%	63%	57%	**63%**
Adverse effects of medical treatment	12%	16%	12%	12%	12%	9%	**12%**
Animal contact (non-venomous)	42%	73%	46%	75%	51%	43%	**53%**
Unintentional injuries not classified elsewhere	51%	68%	49%	58%	54%	49%	**55%**
**Interpersonal violence**	66%	70%	65%	64%	66%	65%	**67%**
**Female**							
**Road injury**	53%	60%	49%	45%	55%	51%	**54%**
**Other transport injury**	76%	73%	72%	61%	73%	72%	**72%**
**Falls**	25%	51%	34%	61%	32%	28%	**36%**
**Fire, heat and hot substances**	40%	39%	28%	31%	37%	37%	**37%**
**Unintentional injury others**							
Exposure to mechanical forces	57%	66%	54%	54%	58%	53%	**58%**
Adverse effects of medical treatment	11%	15%	11%	11%	11%	8%	**11%**
Animal contact (non-venomous)	41%	70%	45%	72%	49%	42%	**52%**
Unintentional injuries not classified elsewhere	44%	60%	43%	51%	48%	43%	**49%**
**Interpersonal violence**	73%	77%	71%	70%	73%	71%	**73%**

2 Extra figures

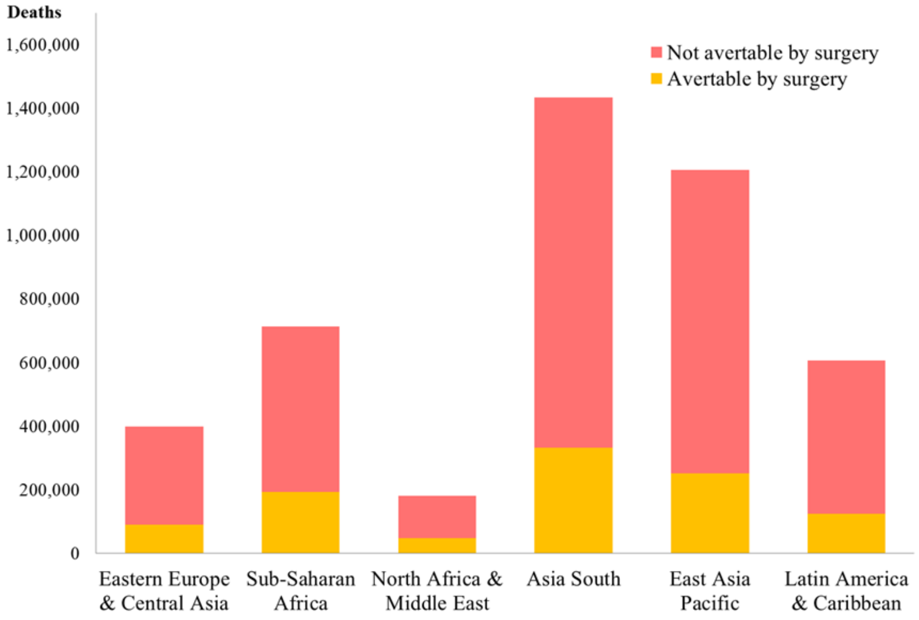

eFigure 1. Surgically avertable and non-avertable deaths from injury in LMICs based on regions

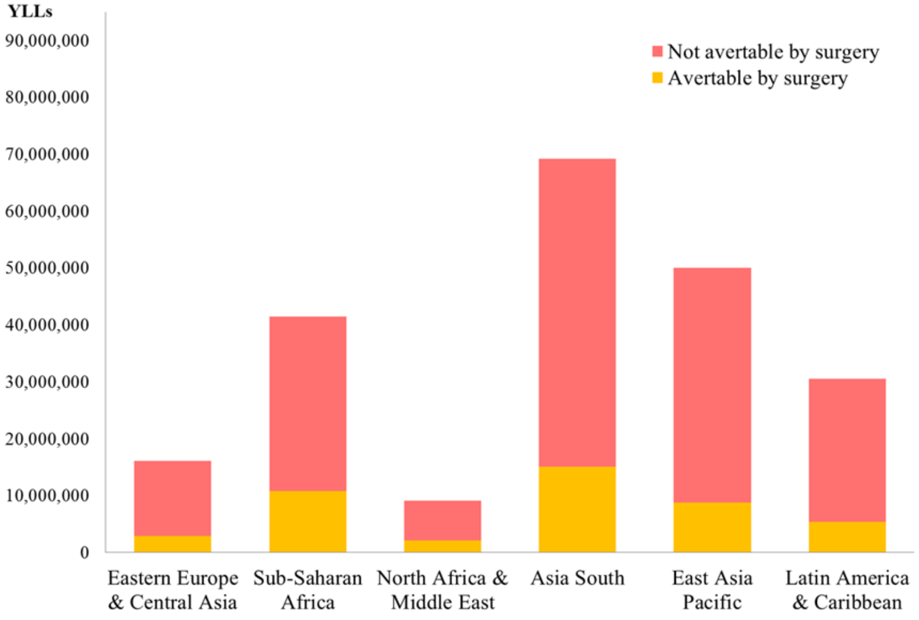

eFigure 2. Surgically avertable and non-avertable YLLs of injury in LMICs based on regions

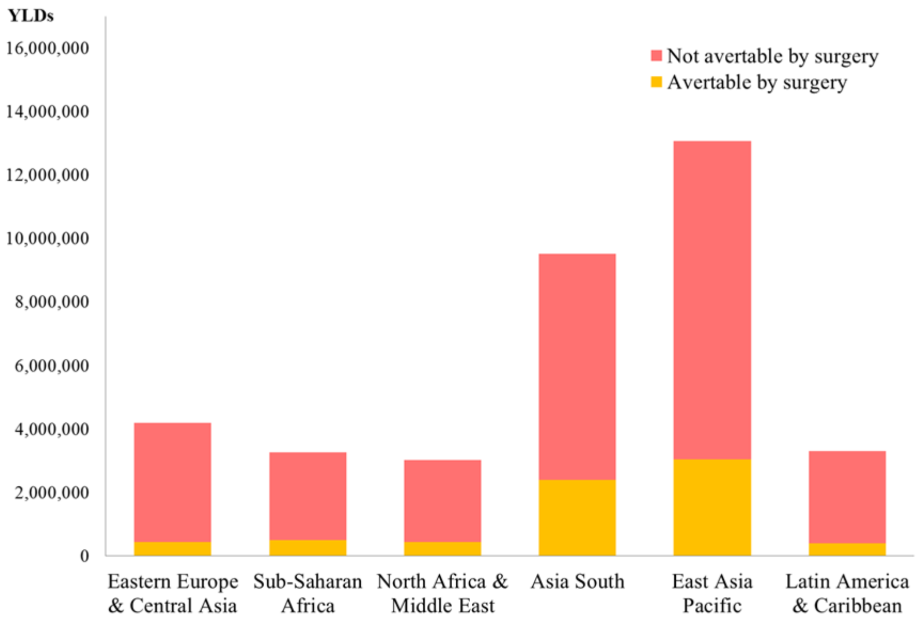

eFigure 3. Surgically avertable and non-avertable YLDs of injury in LMICs based on regions

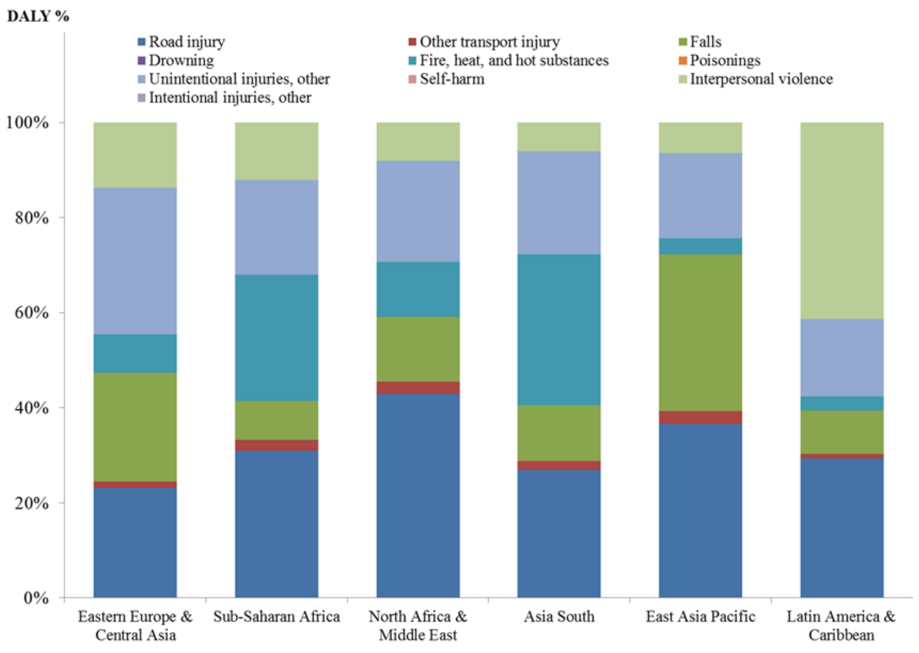

eFigure 4. Proportion of causes of surgically avertable DALYs of injury in each region

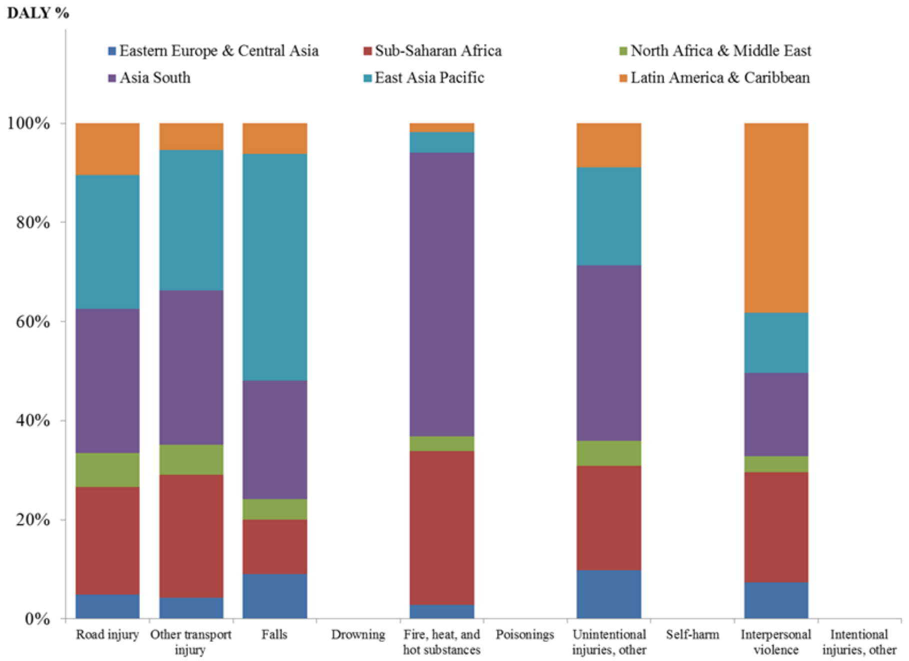

eFigure 5. Proportion of LMIC regions of surgically avertable DALYs of injury for each cause
